# Arsenic and Heavy Metals in Sediments Affected by Typical Gold Mining Areas in Southwest China: Accumulation, Sources and Ecological Risks

**DOI:** 10.3390/ijerph20021432

**Published:** 2023-01-12

**Authors:** Sirui Chen, Pan Wu, Xuefang Zha, Binghuang Zhou, Jingbin Liu, En Long

**Affiliations:** 1 College of Resources and Environmental Engineering, Guizhou University, Guiyang 550025, China; 2 College Key Laboratory of Karst Geological Resources and Environment of Ministry of Education, Guiyang 550025, China

**Keywords:** heavy metals, sediment, gold mining, BCR, PMF, ecological risk

## Abstract

Gold mining is associated with serious heavy metal pollution problems. However, the studies on such pollution caused by gold mining in specific geological environments and extraction processes remain insufficient. This study investigated the accumulation, fractions, sources and influencing factors of arsenic and heavy metals in the sediments from a gold mine area in Southwest China and also assessed their pollution and ecological risks. During gold mining, As, Sb, Zn, and Cd in the sediments were affected, and their accumulation and chemical activity were relatively high. Gold mining is the main source of As, Sb, Zn and Cd accumulation in sediments (over 40.6%). Some influential factors cannot be ignored, i.e., water transport, local lithology, proportion of mild acido-soluble fraction (F_1_) and pH value. In addition, arsenic and most tested heavy metals have different pollution and ecological risks, especially As and Sb. Compared with the other gold mining areas, the arsenic and the heavy metal sediments in the area of this study have higher pollution and ecological risks. The results of this study show that the local government must monitor potential environmental hazards from As and Sb pollution to prevent their adverse effects on human beings. This study also provides suggestions on water protection in the same type of gold-mining areas.

## 1. Introduction

Non-ferrous metal mining causes severe contamination of As and heavy metals (HMs). Due to the increasing activity and mobility in water [1,2], sediments are one of the major destinations for As and HMs migration and diffusion in the water [2,3]. As and HMs accumulated in sediments are often tens or even hundreds of times the amount in water [3,4,5]. The relatively stable and conducive sedimentary environment transforms them into more harmful pollutants or chemical fractions [6]. Once the sedimentary environment is changed by external interference, As and HMs that are accumulated in sediments will be resuspended, which will cause secondary pollution to the water environment [6,7], especially the As and HMs with high mobility that have been affected by the mining area. Therefore, focusing on the accumulation and contamination of As and HMs in sediments around the non-ferrous metal mining area is necessary. Notably, many factors often affect the migration, diffusion, accumulation and release of As and HMs from water to sediment [8,9,10,11].

The heavy metal pollution caused by gold ore should also be paid attention to because of its low grade, and large amount of waste rock and tailings [12]. However, due to the acceleration of industrialization and urban process, extensive mining activities are one of the main reasons for China’s serious environmental problems [13,14]. At present, heavy metal mining pollution and ecological risks are the focus of China’s water and soil protection, especially in the southwest [15,16,17], where special action needs to be urgently conducted due to its large-scale development and utilization of mineral resources, fragile ecological environment and difficulty in recovering after pollution [18,19,20].

Therefore, this study selected a gold mine northeast of Guizhou Province to investigate the arsenic and heavy metals (Sb, Zn, Cd, Cr, Co, Ni, Cu and Pb) in the sediments around the mining area. This gold mine is located in Southwest China, one of the typical gold-mine clusters. It has a mining history of more than 30 years and has affected the local residents to varying degrees [21,22]. It is crucial to identify the source and assess the pollution and risk levels of heavy metals [23,24]. Several receptor models have been proposed over the past decades, such as positive matrix factorization (PMF) that was used in this study, principal component analysis (PCA), and cluster analysis (CA) [25,26]. Metals in the same group are considered to originate from similar sources [25,27,28].

Some key assessment indicators, e.g., the geo-accumulation index (Igeo), potential ecological risk index (RI), and ecological risk factor (Eir), have been confirmed to make important contributions to the ecological risk control of heavy metals. They were also used in this study [29,30]. In addition, some research showed that the fractions of heavy metals in sediments are important for their analysis and evaluation [31]. Hence, this study aimed to (1) clarify the pollution sources of arsenic and heavy metals in sediments, (2) analyze their influencing factors, and (3) assess their pollution and ecological risks to provide a scientific basis and data support for the environmental protection research of the same type of water.

## 2. Materials and Methods

### 2.1. Study Area

The study area is northeast of Southwest Guizhou Province [22]; it has a subtropical monsoon climate, humid and rainy, flat terrain, with an altitude of 1400–1726 m. The annual average temperature is 15.2 °C, and the average precipitation is 1320.5 mm. This area is a typical karst hilly landform, mainly composed of limestone (consisting mainly of calcium carbonate) mixed with a small amount of sandstone and mudstone (in the middle of the study area) and clay rock (in the northeast of the study area).

**Figure 1:**
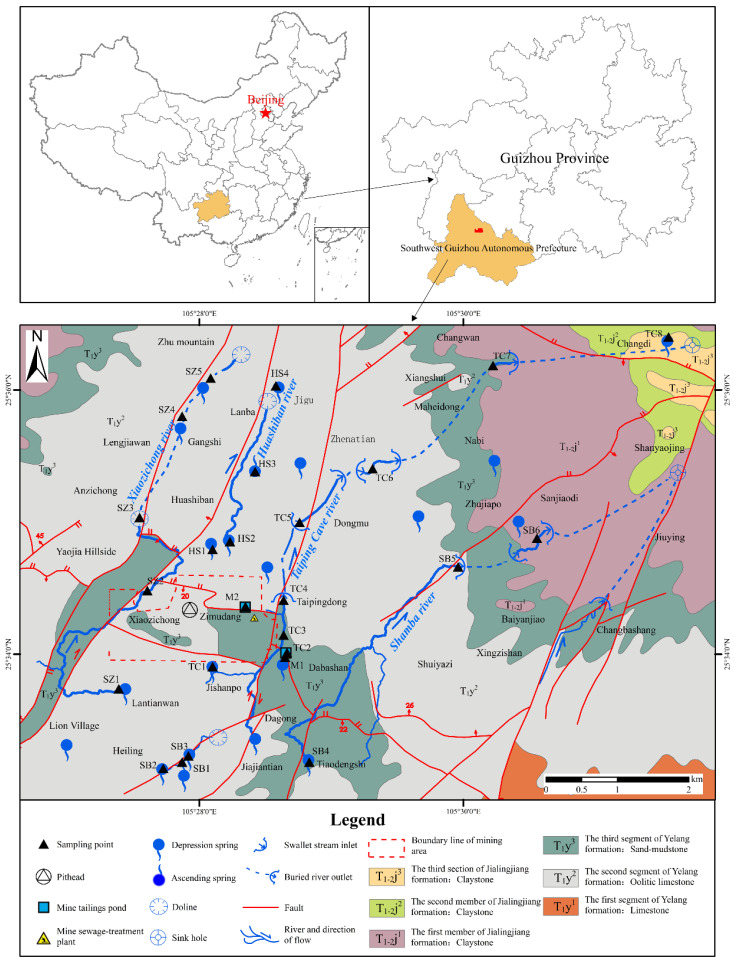
Location, lithology and sampling points of the study area. Except for “Zimudang, Beijing, Guizhou Province, Southwest Guizhou Autonomous Prefecture”, the other place names in the figure represent villages.

The surface water and groundwater in this area are intertwined, and there are four relatively independent karst rivers, Xiaozichong (SZ), Huashiban (HS), Taiping Cave (TC), and Shamba (SB), flowing from southwest to northeast (Figure 1). Gold-bearing minerals are usually pyrite (FeS_2_) and arsenopyrite (AsFeS) [32]. According to the survey, the gold extraction process in this area is mainly Charcoal Immersion Leaching (CIL). In addition to gold mining, cultivation and animal husbandry are the other major activities.

### 2.2. Sample Collection and Analysis

In March 2021, 25 sediment sample points were selected from four karst rivers (SZ, HS, TC and SB), an abandoned mine pit (M_1_), and a mine drainage collection pond (M_2_) in the study area. All samples were collected and uniformly mixed in a single sample point in a polyethylene sealed bag to obtain the final sample of this sample point, which were then sealed and stored at 4 °C and sent for laboratory analysis.

Before testing, the sample was dried in a freeze-dryer (10N-50A, Jingfei Technology, Shenzhen, China) and then screened and impurities were removed. The 1:5 mixed solution of sediment: water (m (g)/V (mL)) was shaken and allowed to stand for 30 min to detect the pH of the sample (phs-3c, Rex instruments, Hangzhou, China). HNO_3_-HF digestion system was selected for hermetic digestion at 180 °C until As and HMs in the sample were completely released, and their concentration was detected. BCR sequential extraction method (GB/T 25282-2010) was selected to extract four chemical forms of As and HMs. An inductively coupled plasma mass spectrometer (ICP-MS, iris intrepid II XSP, Agilent) was used to detect all steps, and the grades of all chemical reagents used were excellent purity.

### 2.3. Quality Control and Statistical Analysis

In this study, the national standard sediment sample (GBW07382 (GSD-31)) and 20% parallel samples were used to ensure the precision and accuracy of the analytical procedure. The results showed that the recoveries of samples were 94.10–113.83%, and the repeatability of parallel samples was 91.10–109.49%. The data were statistically analysed and plotted using Origin 2021 and ArcGIS 10.6 software, and Pearson correlation analysis and source identification were performed using SPSS 20.0 and EPA PMF 5.0.

### 2.4. Background Value of As and HMs, Contamination Assessment Index, and Source Analysis Model

Considering that the regional background value is more appropriate than the average crust or average shale data, the local HMs soil background value of Guizhou Province was adopted as the background value (BV) [33] of the study area. A positive matrix factorization analysis model (PMF) [34] was used to identify the sources of As and HMs accumulation; Assessment of arsenic and HMs pollution and ecological risk based on the geo-accumulation Index (Igeo) [35], single ecological risk factor (Eir) and potential ecological risk index (RI) [36].

## 3. Results

### 3.1. Concentration and Accumulation Changes of As and HMs in Sediments

Tables S1 and S2 (Supplementary Materials) show the concentration and statistical results of As and HMs in sediment samples. In general, except for Pb, the average concentration of As and other HMs was higher than or even much higher than their BV. In contrast, the average concentration of As and HMs in the sediments of the SB karst water system farthest from the mining area was the lowest compared with others, indicating that their input was affected by external activities, among which mining activities in the mining area cannot be ignored.

**Figure 2:**
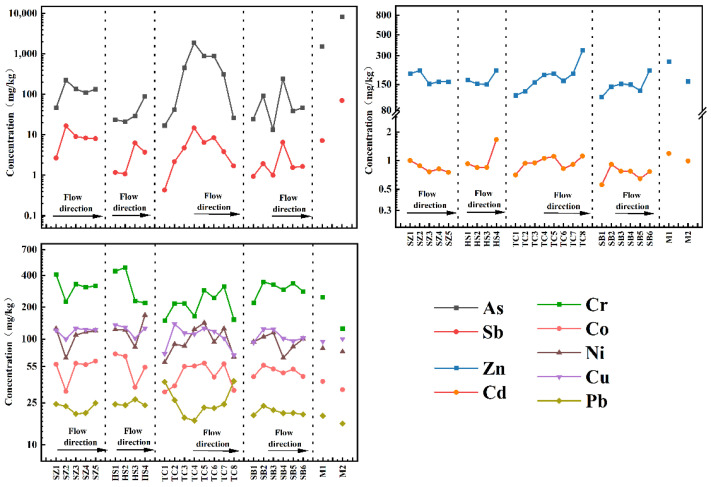
Cumulative changes of As and HMs in the water flow direction of sediments.

Figure 2 shows the cumulative changes of As and HMs in sediment samples with the flow direction. The accumulation changes of ① As and Sb, ② Zn and Cd and ③ Cr, Co, Ni, and Cu in the sediments were similar, indicating that these three groups may have similar accumulation rules and the same accumulation source, respectively. The cumulative degree of As, Sb, Zn and Cd in the mining area (M) and its surrounding sediments (SZ_2_, TC_2_–TC_4_) was very significant, especially the cumulative concentration of As and Sb in M1 and M2 sediments, which was 93.54 and 6.60 times of BV and 408.51 and 31.58 times of BV respectively. Simultaneously, the accumulation of As and Sb in the sediments downstream of the confluence, that is, the intersection of the mining wastewater treatment plant outlet and TC karst river (TC_4_), was also very high, reaching the peak of the TC karst river. This indicates that gold mining seriously impacts the accumulation of As, Sb, Zn and Cd in the sediments, especially As and Sb [37,38,39], similar to the study of other gold deposits [40,41,42,43]. In addition, the As and HMs background in the gold mining area may also be another reason [44].

Overall, there are two phenomena: First, the cumulative concentrations of As, Sb, Zn and Cd in the sediments of the four karst rivers gradually increase with the flow direction. Secondly, the cumulative As and HM concentrations in the sediments at the confluence of all main streams and tributaries (TC_2_ and SB_2_) also increased sharply, indicating that they were affected by water transport because aqueous transport is one of the main ways of migration and diffusion of As and HMs in the dissolved phase [45]. In addition, the accumulation of As, Sb, Zn and Cd in Figure 2 presents a non-unimodal pattern with several prominent concentration turning points, such as TC_5_, TC_6_, SB_4_ and SB_5_, indicating that their accumulation may also be affected by other factors. Compared with As, Sb, Zn and Cd, only part of Cr, Co, Ni and Cu of the sediments slightly decreased around the mining area (SZ_2_ and TC_4_); however, the cumulative characteristics related to the mining area were not evident, which needs further analysis.

### 3.2. Chemical Forms of As and HMs in Sediments

**Figure 3:**
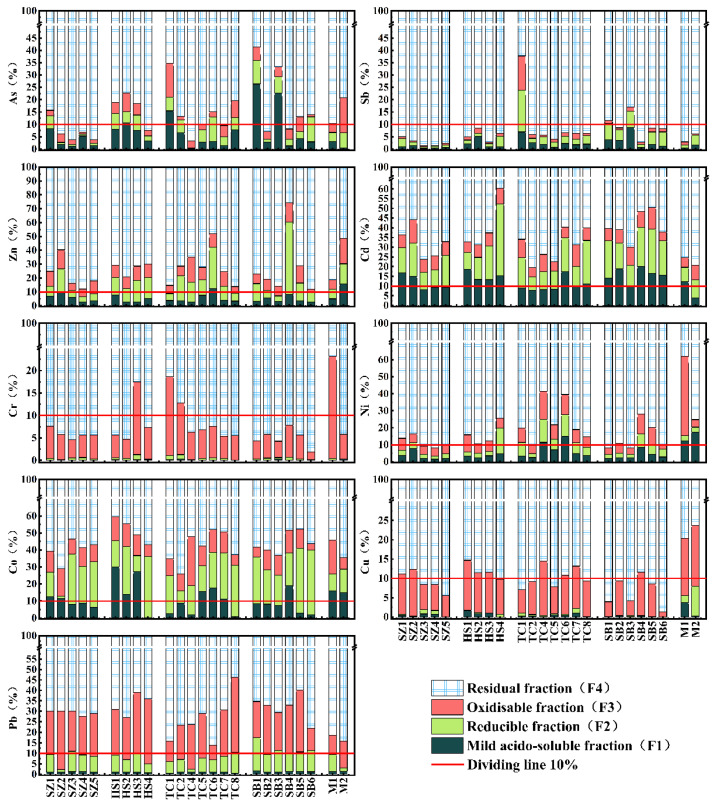
Percentage of four chemical fractions of As and HMs in sediments based on the BCR method.

Figure 3 shows the percentage of the sediment’s four chemical forms of As and HMs. A significant difference in the percentage between As and a single HM was observed. In the non-residual fraction, (1) the percentage of the F_1_ fraction of As, Cd and Co was relatively high, approximately 0–1/3, followed by Sb, Zn and Ni, approximately 0–1/6. However, owing to the strong correlation between the first two HMs, Sb and Zn and aluminum, manganese and iron particles, their fluidity was generally lower than that of other HMs, consistent with this study [46]. On the contrary, Cr, Pb and Cu had almost no F_1_ fraction. Specifically, the F_1_ percentage of As, Sb, Zn and Cd in the sediment was significantly higher than Cr, Co, Ni and Cu; they have higher mobility. Therefore, they can be easily used by aquatic organisms for gold mining, especially since the gold-carrying minerals and associated minerals of this type of gold deposit are rich in a large number of As and Sb [37,38,39], consistent with our previous analysis. (2) Almost all the F_2_ and F_3_ fractions of As and HMs have a certain proportion. Both fractions are vulnerable to the impact of redox fluctuations. They have unstable characteristics, indicating that they have high weak retention in the sediment [47] and are likely to be highly sensitive to some influencing factors that can control or adjust redox conditions. For the residual fractions, (3) the proportion of the F_4_ fraction in the sediment sample was the highest among all fractions, with Cu and Sb being the most prominent (the F_4_ fraction proportion in 98.2% of the sediments exceeded 75%), followed by Ni and As, indicating that the geological background was their non-negligible source, and their solubility reduces to a certain extent [47].

### 3.3. Cumulative Sources of As and HMs in Sediments

**Figure 4:**
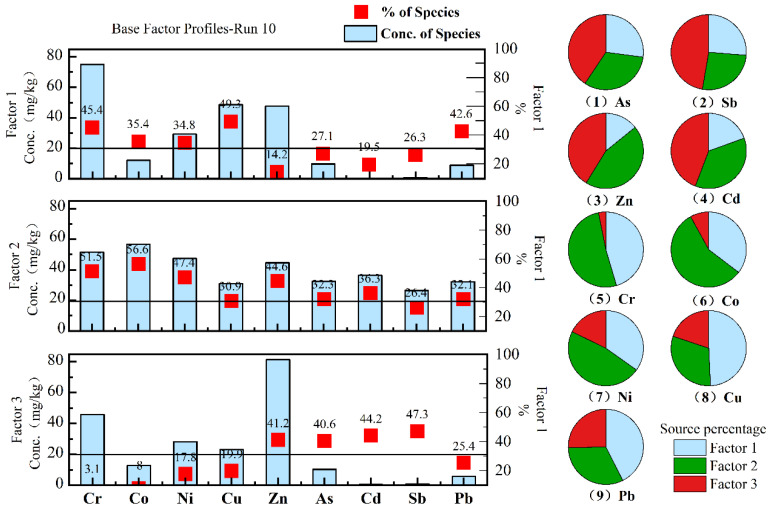
Contribution value, contribution rate and source percentage of As and HMs concentrations in sediments obtained by the PMF model.

Table S3 (Supplementary Materials) shows Pearson correlation coefficients of As and HMs in sediments. There is a significant correlation between these elements (ρ < 0.01), consistent with the analysed results. In the PMF model, the estimated factors are set to 2, 3, 4 and 5, and the predicted and the measured values are fitted, respectively. The fitting results show that it is best when the factor number is 3 because its Q_robust_ (326.4) is closest to Q_ture_ (419.9). Figure 4. respectively show the source contribution and contribution rate of As and HMs in sediments and the percentage of each source when the factor number is 3.

In factor 3, the As, Sb, Zn and Cd had the highest contribution rate (both greater than 40.6%). From the analysis, mining of gold mines has a significant impact on them; therefore, factor 3 is regarded as the source related to it, and the proportion of this part of the source is nearly half in the two groups.

In factor 2, As and HMs have a certain proportion (26.4–56.6%). Therefore, from the percentage of their chemical forms, the geological background is the source of As and all HMs in sediments that cannot be ignored. Concurrently, it is also considered that southwestern China has a background of high concentrations of metalloids and HMs [47,48,49]. Therefore, factor 2 is regarded as a source related to geological background.

In factor 1, the contribution rates of Cr, Co, Ni, Cu and Pb were higher (both greater than 34.8%), while the contribution rates of As, Sb, Zn and Cd were lower (both less than 26.3%). First, we did not observe the cumulative characteristics of Cr, Co, Ni, Cu and Pb related to the mining area. Secondly, compared with As, Sb, Zn and Cd, their chemical activity was lower. Considering that the local villages are evenly distributed (i.e., the area of human activities, Figure 1), these all indicate that they may be more affected by the continuous input of other man-made sources than the gold mining sources, such as domestic sewage. In addition, according to the survey, the local cultivation and animal husbandry are well developed, and some studies show that organic and compound fertilizers contain specific concentrations of Cr, Co, Ni and Cu, especially Cu, which can stimulate plant growth and improve crop yield [50,51,52,53]; thus, agricultural activities may also pollute it. In conclusion, factor 1 is another source mainly based on other human activities. (Because they are not closely related to gold mining, they will not be explained in detail in the following analysis).

### 3.4. Influencing Factors of As and HMs Accumulation in Sediments

In addition to the two factors of gold mining and water transportation, we further discussed the two factors of pH and F_1_ fraction. Concurrently, considering the geological characteristics of karst areas, we also discuss the lithology of this area.

Table S1 and Figure 1 show the lithology of the corresponding area for each sediment. Interestingly, the cumulative changes of As and HMs are very similar to the lithological changes of the sediment’s area. For example, the sediments of several karst rivers (SZ_2_, TC_2_–TC_4_, TC_7_–TC_8_, SB_4_, SB_6_) located in clay rock and sand mudstone areas also show the characteristics of “piecewise” accumulation. Previous studies have shown that colloid flow is the main migration and diffusion of As and HMs besides aqueous transport [11,54,55]. This flow mode is sensitive to the redox state and changes the stability of solid iron (oxygen) oxides, leading to the decomposition of organic matter, thus affecting the migration and diffusion of As and HMs [56]. Notably, As and HMs in almost all sediments in this study showed weak retention and were vulnerable to redox fluctuations (with a high proportion of F_2_ and F_3_ fractions). Studies have shown that lithology can adjust redox fluctuations by influencing soil particle size, mineral composition, and clay content and can indirectly affect the migration and diffusion of As and HMs. In particular, media with high clay content or rich reducible iron (hydroxide) oxides are more easily affected, making As and HMs in the aquatic environment re-accumulate and re-fix [56,57,58].

Therefore, once the As, Sb, Zn and Cd in the water system pass through areas with high clay content (sand mudstone and clay rock in this study), they are very easy to be adsorbed or fixed and finally present high cumulative concentration in the sediments, even in the sediments of non-gold mining areas (SB_4_). This mechanism may also play a role in strengthening the accumulation of As, Sb, Zn and Cd in the sediments of gold mining areas. Considering some As, Sb, Zn and Cd in the upstream are adsorbed or fixed, the cumulative concentration of As and HMs in the downstream sediments (SB_3_, TC_5_ and SB_5_) will be significantly reduced because they have moved from the area with high clay content. In addition, owing to the characteristics of limestone, which can hardly hinder their migration in water, they will continue accumulating to the next area with higher clay content (TC_7_–TC_8_, SB_5_–SB_6_).

**Figure 5:**
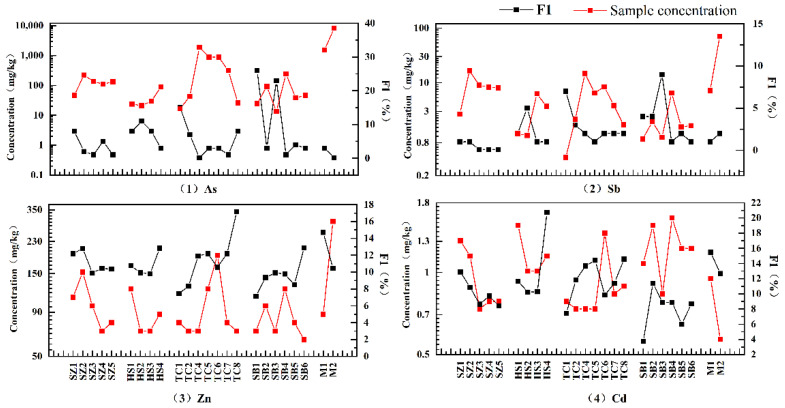
The changing relationship between the percentage change of F1 form and the total amount of As, Sb, Zn and Cd in sediments.

Generally, As and HMs, mainly composed of residue fraction (F_4_), are more stable in the water environment [59,60,61]. However, in this study, the relationship between the percentage change of the F1 fraction in As, Sb, Zn and Cd and the total concentration (Figure 5) shows that there is still a significant negative correlation between them, especially in As and Sb. Furthermore, it shows that As, Sb, Zn and Cd with high mobility can quickly be released from the sediment [62,63], reducing cumulative concentration, even in the sediment of high clay areas (TC_7_–TC_8_).

In addition, limestone (mainly carbonate) also has a certain buffer effect. These stable weak acids, weak alkalis, or neutral pH values can reduce the concentration of soluble As and HMs in karst areas [10]. Most sediment samples in the study may also be affected by this phenomenon because their pH values are neutral or weakly alkaline (7.13–8.86), and only a few sediment samples (SZ_1_, SZ_4_, TB_3 and_ M_1_) are weakly acidic (6.23–6.94).

### 3.5. Contamination and Ecological Risk of As and HMs in Sediments

#### 3.5.1. Contamination and Risk Assessment Based on Igeo, Eir and RI Indexes

**Figure 6:**
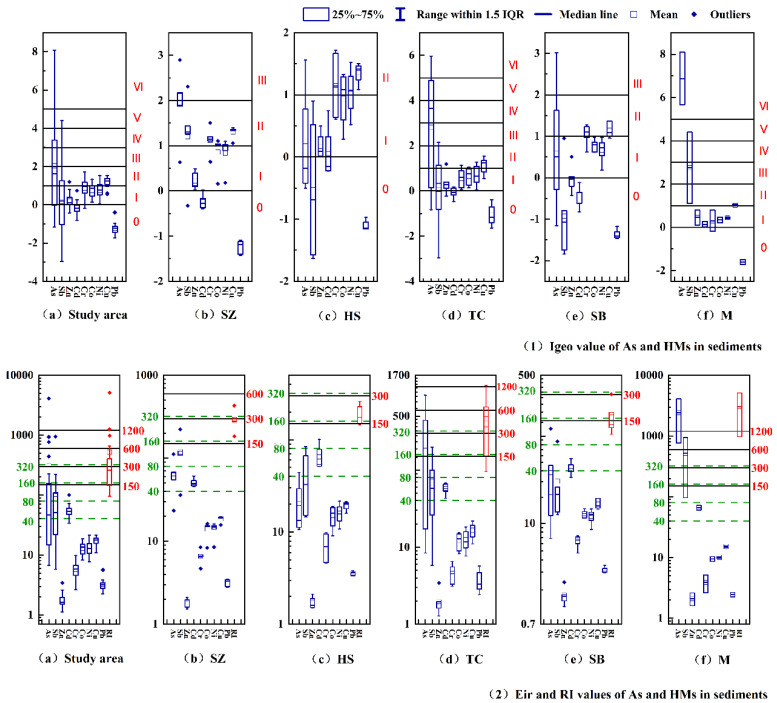
Comparison of Igeo, Eir and RI of As and HMs in sediments of gold mine area and four karst rivers. (**1**) Igeo index: level 0, representing no pollution; Grade I, representing no to moderate pollution; Grade II, representing medium pollution; Grade III, representing moderate to relatively high pollution; Grade IV, representing relatively high pollution to high pollution; Grade V, representing high pollution to extreme pollution; Grade VI, representing extreme pollution. (**2**) Eir index: <40, slight risk; 40–80, medium risk; 80–160, relatively high risk; 160–320, high risk; ≥320, extremely high risk.RI index: <150, slight risk; 150–300, medium risk; 300–600, relatively high risk; 600–1200, high risk; ≥1200, extremely high risk.

Figure 6 shows the comparison of Igeo, Eir and RI of As and HMs in the sediments of the gold mine area and four karst rivers. Igeo and Eir indexes show that the pollution degree of As and other HMs is between grade I and grade II, and the risk level is slight to medium in most sediments except Pb. The contamination degree and risk value of As and Sb were the highest in the sediments; 64% and 4% of the sediments above grade III were polluted, and 24% and 12% of the sediments above high risk were polluted. Notably, as high as 20% of the sediments reached Grade V (severe) pollution, and As and Sb of the extremely high-risk sediments reached 20% and 4%, respectively, and most of them were from the mining area (M) and the TC karst rivers. Especially in the mining area (M_2_), their Eir values were up to 4085.13 and 947.41, respectively, which far exceeded the extremely high-risk standard of the Eir index (Eir > 320). Therefore, we must be alert to their excessive input.

Generally, the average RI index is arranged in descending order: M > CA > SZ ≈ TB > SR, consistent with the previous analysis. Except for Pb, arsenic and other heavy metals are mostly in the range of slight risk to medium risk (12~20%), and 20% and 16.7% of the sediments in SZ and SB rivers were at high risk. However, the sediments above high risk all appeared in TC River and the mining area (M), accounting for 37.5% and 100% of the sediments in their respective water systems, respectively.

#### 3.5.2. Comparison between This Study and Sediments around Other Gold Mining Areas

**Figure 7:**
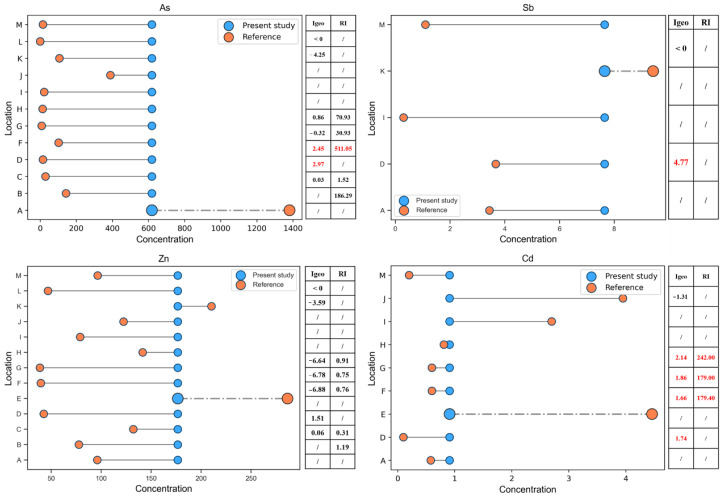
Comparison between the sediments in this study and surface sediments around other gold deposits. Dashed line: represents the value with the largest gap higher than this study’s. Red bold value represents the value exceeding this study. /—Represents missing values in references. A—Orbiel valley (France) [43]. B—Zhaosu River catchment (China) [40]. C—Lom River (Adamawa Cameroon) [64]. D—Anka Gold Mine (Nigeria) [42]. E—Tajum River (Indonesia) [65]. F—Afema Gold Mine (Côte d’Ivoire). G—Agbaou Gold Mine (Côte d’Ivoire). H—Bonikro Gold Mine (Côte d’Ivoire) [41]. I—Gold mine (Gold city, Nigeria) [66]. J—the Black Hills (South Dakota) [67]. K—Santurbán paramo (Colombia) [68]. L—Itapicuru-Mirim River (Brazil) [69]. M—Gold mine (Kesennuma City, Japan) [70].

The average concentration, Igeo and RI values of As, Sb, Zn and Cd in the sediments of this study were compared with the surface sediments around other gold mining areas (Figure 7).

The average concentrations of As and HMs in the sediments of most references are lower than those in our study. Individual average values, including As, are extremely low relative to local A, possibly owing to their different gold ore types or mining methods because the high concentration of As in this area is concentrated near the mining area. Sb is lower than local K. Zn is lower than local K and E, and the reason for Zn being lower than local E is the same as above. Cd is lower than local E, I and J. For Igeo and RI values, Zn in other studies is lower than that in our study. On the contrary, Cd is mostly higher than that in our research. However, there are two evaluation criteria in these cities: (1) A, B, D, K and M were evaluated by statistical methods or local background values. (2) C, F, G, H and L were evaluated by the geochemical background value of the crust (UCC). Most of the Igeo and RI values higher than those in our study were evaluated using UCC as the background value, except for area D. Notably, the background value of UCC was far lower than that of this study. This means that the pollution and ecological risks of arsenic and heavy metals in the study area are still relatively serious and must be paid significant attention.

## 4. Conclusions

This study analyzed the accumulation, sources and impact factors and assessed their pollution and ecological risks of As and HMs in the river sediments around a gold mine in southwest China, and obtained some important results: (1) Compared with Cr, Co, Ni, Cu and Pb, As, Sb, Zn and Cd were affected by gold mining, and their accumulation degree and chemical activity were relatively high. Among them, the sediments with high As and Sb accumulation were mainly concentrated in the gold mine area (M). The cumulative concentrations of As, Sb, Zn and Cd in the sediments of the SZ, HS and TC karst rivers were higher than those of the SB karst rivers. (2) Gold mining is the primary source of As, Sb, Zn and Cd accumulation in sediments, accounting for 40.6%, 47.3%, 41.2% and 44.2%, respectively. In addition, water flow transport, local lithology, the proportion of F1 fraction of elements, and pH are influencing factors that cannot be ignored. (3) In addition to Pb, arsenic and other heavy metals have reached a slight to medium level of pollution and risk. However, As and Sb are the most serious. Their Igeo and Eir values are above serious pollution and medium pollution, respectively, and both meet extremely high-risk standards, mainly the sediments from the mining area (M) and TC River. The research result is of great significance for preventing and controlling the sediment pollution of arsenic and heavy metals near the same type of gold mining area. In addition, other influencing factors, such as local lithology and weak acid distillate, should be noted.

## Data Availability

Some or all data, models, or code that supports the findings of this study is available from the corresponding author upon reasonable request.

## Supplementary Materials

The following supporting information can be downloaded at: https://www.mdpi.com/article/10.3390/ijerph20021432/s1, Table S1. As and HMs concentration (mg/kg), pH value in sediment samples, and lithology of the area. Table S2. Statistical results of concentration (mg/kg) and pH of As and HMs in sediment samples and their corresponding BV. Table S3. Pearson correlation matrix of As and HMs in sediments.
