# Supplementary material for: Arsenic and Heavy Metals in Sediments Affected by Typical Gold Mining Areas in Southwest China: Accumulation, Sources and Ecological Risks
Source: Int J Environ Res Public Health. 2023 Jan 12;20(2):1432. doi: 10.3390/ijerph20021432 (PMC9859441; doi:10.3390/ijerph20021432)
Supplement: Supplementary file 1 [file ijerph-20-01432-s001.zip › ijerph-2140901-supplementary.pdf]

**Supplementary Materials**

Accumulation and ecological risk assessment of arsenic and heavy metals in sediments affected by gold mining in karst areas of Southwest China

Sirui Chen[a,b], Pan Wu[a,b,*], Xuefang Cha[a,b], Binghuang Zhou[a,b], Jingbin Liu[a,b], En Long[a,b]

[a] College of Resources and Environmental Engineering, Guizhou University, Guiyang 550025, China
[b] College Key Laboratory of karst geological resources and environment of Ministry of education, Guiyang 550025, China

* Corresponding author: Pan Wu
Email: pwu@gzu.edu.cn

**The following supplementary material is available for this article online:**

**Table S1.** As and HMs concentration (mg/kg), pH value in sediment samples, and lithology of the area.

**Table S2.** Statistical results of concentration (mg/kg) and pH of As and HMs in sediment samples and their corresponding BV.

**Table S3.** Pearson correlation matrix of As and HMs in sediments.

**Table S1. As and HMs concentration (mg/kg), pH value in sediment samples, and lithology of the area.**

| Sample number | Lithology of the area | Cr | Co | Ni | Cu | Zn | As | Cd | Sb | Pb | pH |
|---|---|---|---|---|---|---|---|---|---|---|---|
| | | | | | **SZ** | | | | | | |
| SZ1 | Oolitic limestone | 407.51 | 57.78 | 125.43 | 121.18 | 195.42 | 46.54 | 1.00 | 2.68 | 24.26 | 6.94 |
| SZ2 | Sand-mudstone | 224.53 | 32.05 | 66.30 | 99.85 | 209.56 | 223.17 | 0.88 | 16.62 | 23.18 | 7.03 |
| SZ3 | Oolitic limestone | 329.90 | 59.01 | 109.01 | 126.04 | 151.49 | 135.15 | 0.76 | 9.06 | 19.59 | 7.16 |
| SZ4 | Oolitic limestone | 309.76 | 57.20 | 116.32 | 123.20 | 160.82 | 109.91 | 0.82 | 8.31 | 19.97 | 6.94 |
| SZ5 | Oolitic limestone | 317.09 | 61.86 | 119.57 | 122.83 | 159.68 | 134.09 | 0.75 | 8.06 | 24.78 | 7.55 |
| | | | | | **HS** | | | | | | |
| HS1 | Oolitic limestone | 439.06 | 72.27 | 123.10 | 136.13 | 166.62 | 23.66 | 0.93 | 1.18 | 24.19 | 7.13 |
| HS2 | Oolitic limestone | 473.69 | 68.72 | 121.67 | 128.96 | 151.90 | 21.23 | 0.85 | 1.08 | 23.70 | 7.23 |
| HS3 | Oolitic limestone | 228.75 | 35.07 | 83.91 | 101.98 | 149.51 | 29.60 | 0.85 | 6.28 | 26.90 | 6.94 |
| HS4 | Oolitic limestone | 219.60 | 54.13 | 168.32 | 126.53 | 210.40 | 88.34 | 1.66 | 3.71 | 23.65 | 7.58 |
| | | | | | **TC** | | | | | | |
| TC1 | Oolitic limestone | 149.20 | 31.59 | 60.34 | 72.68 | 175.10 | 16.81 | 0.71 | 0.43 | 39.37 | 7.74 |
| TC2 | Sand-mudstone | 216.80 | 36.13 | 89.49 | 138.97 | 126.94 | 42.42 | 0.94 | 2.16 | 26.50 | 7.4 |
| TC3 | Sand-mudstone | 217.76 | 55.05 | 85.83 | 113.90 | 158.30 | 456.73 | 0.95 | 4.75 | 18.05 | 7.52 |
| TC4 | Sand-mudstone | 164.83 | 55.59 | 123.55 | 111.65 | 189.85 | 1870.80 | 1.06 | 14.79 | 16.93 | 7.92 |
| TC5 | Oolitic limestone | 290.08 | 59.31 | 142.26 | 126.19 | 195.34 | 884.23 | 1.11 | 6.47 | 22.53 | 7.18 |
| TC6 | Oolitic limestone | 243.70 | 43.72 | 93.69 | 118.29 | 163.33 | 885.58 | 0.83 | 8.49 | 22.19 | 7.2 |
| TC7 | Claystone | 313.50 | 58.35 | 125.27 | 101.84 | 195.37 | 312.09 | 0.91 | 3.84 | 24.20 | 7.66 |
| TC8 | Claystone | 152.58 | 32.75 | 67.87 | 70.44 | 341.72 | 26.11 | 1.12 | 1.70 | 40.14 | 7.97 |

**Table S1.（Continued）As and HMs concentration (mg/kg), pH value in sediment samples, and lithology of the area.**

| Sample number | Lithology of the area | Cr | Co | Ni | Cu | Zn | As | Cd | Sb | Pb | pH |
|---|---|---|---|---|---|---|---|---|---|---|---|
| | | | | | **SB** | | | | | | |
| SB1 | Oolitic limestone | 220.59 | 44.08 | 94.01 | 92.81 | 111.19 | 24.62 | 0.56 | 0.94 | 19.10 | 7.35 |
| SB2 | Oolitic limestone | 347.52 | 56.42 | 105.02 | 123.96 | 142.42 | 92.41 | 0.91 | 1.94 | 23.35 | 8.68 |
| SB3 | Oolitic limestone | 325.54 | 52.16 | 114.79 | 123.57 | 151.58 | 13.46 | 0.78 | 1.00 | 21.30 | 7.27 |
| SB4 | Sand-mudstone | 292.19 | 47.81 | 66.46 | 101.12 | 149.38 | 242.78 | 0.78 | 6.51 | 19.94 | 7.44 |
| SB5 | Oolitic limestone | 336.89 | 51.94 | 84.75 | 95.65 | 129.56 | 38.43 | 0.65 | 1.56 | 20.00 | 7.32 |
| SB6 | Claystone | 282.24 | 44.20 | 100.50 | 102.10 | 211.28 | 46.83 | 0.77 | 1.65 | 19.40 | 7.14 |
| | | | | | **M** | | | | | | |
| M1 | Sand-mudstone | 249.66 | 39.75 | 81.56 | 94.89 | 259.42 | 1530.83 | 1.19 | 7.21 | 18.80 | 6.23 |
| M2 | Sand-mudstone | 125.77 | 33.31 | 75.96 | 100.56 | 160.91 | 8170.25 | 0.99 | 70.74 | 15.90 | 8.39 |

**Table S2. Statistical results of concentration (mg/kg) and pH of As and HMs in sediment samples and their corresponding BV.**

| | **Cr** | **Co** | **Ni** | **Cu** | **Zn** | **As** | **Cd** | **Sb** | **Pb** | **pH** |
|---|---|---|---|---|---|---|---|---|---|---|
| | | | | | **SZ** | | | | | |
| Min | 224.53 | 32.05 | 66.30 | 99.85 | 151.49 | 46.54 | 0.75 | 2.68 | 19.59 | 6.94 |
| Max | 407.51 | 61.86 | 125.43 | 126.04 | 209.56 | 223.17 | 1.00 | 16.62 | 24.78 | 7.55 |
| Mean±SD | 317.76±65.11 | 53.58±12.17 | 107.33±23.69 | 118.62±10.64 | 175.39±25.49 | 129.77±63.43 | 0.84±0.10 | 8.95±4.98 | 22.36±2.43 | 7.12±0.25 |
| | | | | | **HS** | | | | | |
| Min | 219.60 | 35.07 | 83.91 | 101.98 | 149.51 | 21.23 | 0.85 | 1.08 | 23.65 | 6.94 |
| Max | 473.69 | 72.27 | 168.32 | 136.13 | 210.40 | 88.34 | 1.66 | 6.28 | 26.90 | 7.58 |
| Mean±SD | 340.28±134.86 | 57.55±16.91 | 124.25±34.53 | 123.40±14.85 | 169.61±28.23 | 40.71±31.95 | 1.07±0.39 | 3.06±2.46 | 24.61±1.55 | 7.22±0.27 |
| | | | | | **TC** | | | | | |
| Min | 149.20 | 31.59 | 60.34 | 70.44 | 126.94 | 16.81 | 0.71 | 0.43 | 16.93 | 7.18 |
| Max | 313.50 | 59.31 | 142.26 | 138.97 | 341.72 | 1870.80 | 1,12 | 14.79 | 40.14 | 7.97 |
| Mean±SD | 218.56±61.89 | 46.56±11.87 | 98.54±29.08 | 106.75±24.28 | 193.24±64.26 | 561.85±638.01 | 0.95±0.14 | 5.33±4.64 | 26.24±8.89 | 7.57±0.30 |
| | | | | | **SB** | | | | | |
| Min | 220.59 | 43.10 | 66.46 | 92.81 | 93.69 | 13.46 | 0.56 | 0.94 | 19.10 | 7.14 |
| Max | 347.52 | 56.42 | 114.79 | 123.96 | 211.28 | 242.78 | 0.98 | 6.51 | 29.02 | 8.68 |
| Mean±SD | 300.83±46.80 | 49.44±4.92 | 94.26±16.26 | 106.54±13.78 | 149.24±33.87 | 76.42±85.90 | 0.74±0.12 | 2.27±2.11 | 20.52±1.58 | 7.53±0.57 |
| | | | | | **M** | | | | | |
| M1 | 249.66 | 39.75 | 81.56 | 94.89 | 259.42 | 1530.83 | 1.19 | 7.21 | 18.80 | 6.23 |
| M2 | 125.77 | 33.31 | 75.96 | 100.56 | 160.91 | 8170.25 | 0.99 | 70.74 | 15.90 | 8.39 |
| BV | 95.9 | 19.2 | 39.1 | 32.0 | 99.5 | 20.0 | 0.66 | 2.24 | 35.20 | |
| Mean（All）±SD | 273.15±88.47 | 49.61±11.77 | 101.80±26.50 | 111.01±17.95 | 176.68±47.28 | 618.64±1646.78 | 0.91±0.22 | 7.65±13.82 | 23.12±5.77 | 7.39±0.50 |

BV: HMs soil background value of Guizhou Province

**Table S3. Pearson correlation matrix of As and HMs in sediments**

| | Cr | Co | Ni | Cu | Zn | As | Cd | Sb | Pb |
|---|---|---|---|---|---|---|---|---|---|
| **Cr** | 1 | | | | | | | | |
| **Co** | 0.791★★ | 1 | | | | | | | |
| **Ni** | 0.389 | 0.701★★ | 1 | | | | | | |
| **Cu** | 0.535★★ | 0.632★★ | 0.654★★ | 1 | | | | | |
| **Zn** | -0.291 | -0.222 | -0.018 | -0.427 | 1 | | | | |
| **As** | -0.418 | -0.279 | -0.164 | -0.095 | 0.006 | 1 | | | |
| **Cd** | -0.208 | 0.096 | 0.532★ | 0.245 | 0.548★ | 0.151 | 1 | | |
| **Sb** | -0.421 | -0.296 | -0.176 | -0.088 | -0.042 | 0.989★★ | 0.130 | 1 | |
| **Pb** | -0.190 | -0.348 | -0.263 | -0.404 | 0.430 | -0.377 | 0.033 | -0.355 | 1 |

★Correlated： $p < 0.05$

★★Significantly correlated： $p < 0.01$
